# Attitudes of Neurologists Toward Serum Neurofilament Light-Chain Testing in the Management of Relapsing–Remitting Multiple Sclerosis with Cognitive Impairment

**DOI:** 10.3390/jpm15020069

**Published:** 2025-02-14

**Authors:** José M. García-Domínguez, Jorge Maurino, José E. Meca-Lallana, Lamberto Landete, Virginia Meca-Lallana, Elena García-Arcelay, Eduardo Agüera-Morales, Ana B. Caminero, Sergio Martínez-Yélamos, Luis Querol, Nicolas Medrano, Rocío Gómez-Ballesteros, Luisa M. Villar, Enric Monreal, Gustavo Saposnik

**Affiliations:** 1 Department of Neurology, Hospital Universitario Gregorio Marañón, 28007 Madrid, Spain; 2 Medical Department, Roche Farma, 28042 Madrid, Spain; elena.garcia_arcelay.eg1@roche.com (E.G.-A.); nicolas.medrano@roche.com (N.M.); rocio.gomez@roche.com (R.G.-B.); 3 Department of Neurology, Hospital Clínico Universitario Virgen de la Arrixaca, 30120 Murcia, Spain; pmecal@gmail.com; 4 Department of Neurology, Hospital Universitario Dr. Peset, 46017 Valencia, Spain; lamberto676@gmail.com; 5 Department of Neurology, Hospital Universitario La Princesa, 28006 Madrid, Spain; virmeca@hotmail.com; 6 Maimonides Biomedical Research Institute of Cordoba (IMIBIC), Reina Sofia University Hospital, University of Cordoba, 14004 Córdoba, Spain; doctoredu@gmail.com; 7 Department of Neurology, Complejo Asistencial de Ávila, 05071 Ávila, Spain; caminero.nrl@gmail.com; 8 Department of Neurology, Hospital Universitari de Bellvitge, L’Hospitalet de Llobregat, 08907 Barcelona, Spain; smartinezy@bellvitgehospital.cat; 9 Department of Neurology, Hospital de la Santa Creu i Sant Pau, 08025 Barcelona, Spain; lquerol@santpau.cat; 10 Department of Immunology, Hospital Universitario Ramón y Cajal, 28034 Madrid, Spain; luisamaria.villar@salud.madrid.org; 11 Department of Neurology, Hospital Universitario Ramón y Cajal, 28034 Madrid, Spain; enricmonreal@outlook.com; 12 Division of Neurology, Department of Medicine, St. Michael’s Hospital, University of Toronto, Toronto, ON M5B 1W8, Canada; gustavo.saposnik@unityhealth.to

**Keywords:** neurofilament light chain, cognition, multiple sclerosis, decision-making, personalized medicine

## Abstract

**Background:** Cognitive impairment has an impact upon the function and quality of life of patients with multiple sclerosis (MS). High-serum neurofilament light-chain (sNfL) levels predict disease progression and are also associated with impaired cognitive performance. This study aimed to assess the attitudes of neurologists toward sNfL testing as regards making therapeutic decisions in clinically and radiologically stable patients experiencing cognitive decline. **Methods:** A web-based observational study was conducted among neurologists caring for patients with MS. The role of sNfL in therapeutic decisions was assessed through a simulated case scenario describing a 31-year-old woman with relapsing–remitting MS for four years on glatiramer acetate. Her partner reported increased distractibility and difficulties in organizing daily activities over the past 18 months. There was no history of new relapses, and a follow-up brain MRI scan showed no new lesions. Her performance in the Symbol Digit Modalities Test decreased by 8 points from the previous year, with 46 correct answers. The patient had an sNfL level of 21 pg/mL, with no other identified factors that could have altered this value. The participants were tasked with deciding to either escalate treatment or to continue the current treatment and schedule the patient for reassessment in 6–12 months (defined as decisions misaligned with emerging evidence [DMEE]). Multivariate regression analysis was conducted to determine factors associated with DMEE. **Results:** One hundred and sixteen neurologists participated in the study. Almost 50% of the participants (*n* = 57) opted not to escalate treatment despite high sNfL levels. This was more common among neurologists not fully dedicated to MS care (60.5% vs. 43.6%). The multivariate analysis showed that being a neurologist not fully dedicated to MS (odds ratio [OR] = 2.35, 95% confidence interval [CI] 1.01–5.50; *p* = 0.04) and having a poor perception of sNfL benefits (OR = 1.02, 95% CI 1.00–1.04; *p* = 0.01) were associated with DMEE. **Conclusions:** Neurologists’ lack of full dedication to MS care and limited perception of sNfL’s clinical utility were key factors associated with suboptimal therapeutic decisions in a simulated case of cognitive decline with elevated sNfL. These findings underscore the need for increased education on the role of sNfL to improve evidence-based decision-making in MS management.

## 1. Introduction

Multiple sclerosis (MS) is a chronic autoimmune disorder that targets the central nervous system, leading to neurodegeneration [1]. It predominantly affects young, active individuals. The most common clinical form is relapsing–remitting MS (RRMS), which is characterized by recurrent episodes of neurological symptoms such as vision problems, muscle weakness, coordination and balance issues, and numbness. These episodes are followed by periods of stability in which the symptoms may partially or fully subside, or by residual impairment.

Among patients with RRMS, cognitive impairment is reported in 30–45% of cases, predominantly impacting processing speed, memory, and executive function [2,3,4]. Major risk factors include a later onset of the disease, higher levels of disability, cardiovascular risk factors, and reduced physical activity during childhood and adolescence [5]. Notably, in most cases, cognitive decline occurs independently of disease relapses or Expanded Disability Status Scale progression, significantly impacting quality of life and employment [3,6,7]. Cognitive problems are also frequently observed as patients transition to the secondary progressive phase of the disease [2]. This phase is characterized by a gradual accumulation of disability, often accompanied by a worsening of cognitive function.

The neurofilament light chain, a key intermediate cytoskeletal protein found in axons, is regarded as a reliable biomarker for axonal damage in several neurodegenerative and inflammatory disorders [8]. In MS care, serum neurofilament light chain (sNfL) testing used to forecast the long-term prognosis and assess treatment efficacy [9,10,11]. Elevated sNfL levels are also associated with cognitive impairment, particularly slower processing speed, and are a predictor of future cognitive decline [12,13,14,15,16,17].

The management of cognitive impairment in MS patients remains a contentious issue in the field [3,18,19,20]. While early escalation therapy is often proposed to mitigate further neurological damage, its long-term impact on cognitive outcomes and safety profiles is not fully established. Conversely, a conservative approach may risk delaying necessary interventions. The challenge lies in accurately identifying patients who are most likely to benefit from escalation therapy, especially when cognitive decline occurs independently of overt relapses or EDSS worsening.

The early initiation of high-efficacy disease-modifying therapies has been linked to more significant improvements in cognitive function compared to delayed treatment or the use of first-line therapies [18,19,20]. However, there are no specific guidelines explicitly stating that cognitive impairment alone should prompt a change in disease-modifying treatment [2,3,21]. In this context, the availability of a reliable and noninvasive biomarker of neuroaxonal damage, such as sNfL, could assist neurologists in making personalized therapeutic decisions [11]. The objective of this study was to assess the attitudes of neurologists toward sNfL testing in the management of RRMS with cognitive impairment.

## 2. Methods

A web-based observational study was conducted by neurologists who provide care to patients with MS. Neurologists caring for patients with MS were invited to participate in the survey via e-mail by the Spanish Society of Neurology. The study was conducted according to the guidelines of the Declaration of Helsinki, and approved by the Institutional Review Board of Hospital Universitario Clínico San Carlos in Madrid, Spain (protocol code: 23/471-E). Informed consent was obtained from all subjects involved in the study.

Participants were asked to respond to different management options presented through a simulated case scenario describing a hypothetical patient with RRMS, a pathological Symbol Digit Modalities Test (SDMT) score, and elevated sNfL levels. Demographic characteristics, professional background, clinical setting, behavioral traits, and expectations regarding the usefulness of sNfL testing were also collected.

Participants were exposed to the following case scenario: “A 31-year-old woman with relapsing–remitting multiple sclerosis, diagnosed four years ago and currently on glatiramer acetate treatment, has been experiencing cognitive decline. Her partner reports increased distractibility and difficulty in organizing and planning daily activities over the past year and a half. She has decreased by 8 points on the Symbol Digit Modalities Test (SDMT) since her last evaluation a year ago, with a current score of 46 correct responses (SDMT pathological cutoff score ≤ 49). She does not exhibit any mood disorders. A follow-up brain magnetic resonance imaging scan shows no new lesions and her sNfL levels are 21 pg/mL. There are no other factors found that could have altered the value of sNfL”. Participants were asked to decide whether to schedule her for re-evaluation in 6 to 12 months, including a neuropsychological assessment, or to switch her treatment to a more effective disease-modifying therapy. The following treatment options were presented: fingolimod, ocrelizumab, and natalizumab. Participants were explicitly instructed at the beginning of the exercise as follows: “We invite you to participate in a short survey to understand how sNfL testing influences decisions in this case. The case is presented as a hypothetical scenario where decisions should not be conditioned by hospital protocols, drug access, or costs. Responses should reflect your clinical judgment only. The pathological cut-off point for sNfL levels will be 10 pg/mL”.

The primary outcome that emerged was decisions misaligned with emerging evidence (DMEE), defined as a failure to intensify treatment despite evidence of cognitive decline and elevated sNfL levels. This definition was adapted from previous studies assessing treatment choices in MS [22,23].

### 2.1. Measurements

The Evidence-Based Practice Attitude Scale (EBPAS), the Jefferson Scale of Empathy (JSE-HP), the Regret Intensity Scale (RIS-10), and a single item from the Physician Work Life Study were used to collect information on attitudes toward innovations, medical empathy, healthcare-related regret, and occupational burnout [24,25,26,27]. The scoring methods and range of each scale are presented in Table 1.

### 2.2. Statistical Analysis

Logistic regression models were used to assess the relationship between participants’ characteristics and the primary binary outcome of interest: DMEE. The adjusted analysis included the following explanatory variables based on conceptual and clinical relevance: age, sex, years of professional practice, experience managing MS patients, the type of hospital (academic vs. non-academic), full or partial dedication to MS care, the weekly volume of patients, the availability of sNfL testing, involvement in MS clinical trials, occupational burnout, empathy levels, openness to innovation, and feelings of care-related regret. All variables were included simultaneously in the multivariable model to assess their independent associations with DMEE.

## 3. Results

One hundred and sixteen neurologists participated in the study. The mean age (SD) was 41.9 (10.1) years, and 53.4% of participants were male. The majority of participants (96.6%) worked in academic hospitals and had a mean of 12.6 (8.1) years of experience in caring for MS patients. Table 1 shows the characteristics of participants.

**Table 1:** Main characteristics of participants.

Characteristics	*n* = 116
Age, years, mean (SD)	41.9 (10.1)
Sex, male, *n* (%)	62 (53.4)
Years as neurologist, mean (SD)	16.0 (9.2)
Years managing MS patients, mean (SD)	12.6 (8.1)
Institution type, academic, *n* (%)	112 (96.6)
Weekly MS patients load, median (IQR)	16.0 (10.0, 25.0)
Fully dedicated to MS care, *n* (%)	78 (67.2)
Access to measurement of sNfL, *n* (%)	40 (34.5)
Investigator in MS clinical trials, *n* (%)	63 (54.3)
EBPAS score ^1^ (range: 0–4), mean (SD)	2.8 (0.4)
JSE-HP score ^2^ (range: 20–140), mean (SD)	107.7 (12.2)
Occupational burnout ^3^, *n* (%)	33 (28.4)
RIS-10 score ^4^ (range: 1–5), mean (SD)	2.1 (0.8)

EBPAS, Evidence-Based Practice Attitude Scale; IQR, Interquartile range; JSE-HP, Jefferson Scale of Empathy—Health Professionals; MS, multiple sclerosis; RIS-10, Regret Intensity Scale; SD, standard deviation; sNfL, serum neurofilament light chain. ^1^ higher scores indicate a favorable attitude towards healthcare innovations. ^2^ higher scores indicate greater empathy toward patients. ^3^ cutoff score ≥ 3. ^4^ higher scores indicate a greater intensity of regret related to a past experience in caring for a patient.

Overall, 49.1% of the participants (*n* = 57) chose not to escalate treatment, even when presented with evidence of cognitive decline and elevated sNfL levels. This finding was more common among neurologists not fully dedicated to MS care (60.5% vs. 43.6%; *p* = 0.09). The multivariate analysis showed that being a neurologist not fully dedicated to MS care (odds ratio [OR] = 2.40, 95% confidence interval [CI] 1.03–5.57; *p* = 0.04) and having a poor perception of sNfL benefits (OR = 1.02, 95% CI 1.00–1.04; *p* = 0.016) were associated with DMEE. The demographic and behavioral characteristics of the participants, the type of hospital, the volume of patients, and availability of sNfL testing were not associated with DMEE (Table 2).

## 4. Discussion

Cognitive decline is a significant aspect of MS that affects daily functioning, work, social interactions, and mental health, often progressing independently of physical disability and relapses [2,3,6]. A recent retrospective study involving 336 patients with RRMS found that 50.3% experienced cognitive impairment over an 8-year follow-up period [28]. The sample had a mean age of 43.1 years and a mean disease duration of 10.8 years. Notably, cognitive decline occurred independently of relapses in 89% of cases and without EDSS worsening in 68.1%.

Escalation strategies, which begin with moderate-efficacy disease-modifying therapies and transition to higher-efficacy options if disease activity persists, have traditionally been the standard treatment approach for patients with RRMS [29]. However, initiating a higher-efficacy disease-modifying therapy promptly after diagnosis is becoming increasingly common as an alternative method with which to mitigate long-term disability. Despite these advancements, addressing cognitive decline remains a significant challenge for neurologists [3,18,30]. High-efficacy disease-modifying therapies have been shown to significantly enhance cognitive functions, particularly in terms of processing speed, as assessed by the SDMT [18,20]. Research indicates that treatments like alemtuzumab and ocrelizumab lead to clinically significant gains in SDMT scores, with a percentage of patients experiencing cognitive improvements [20]. For instance, 60% of the patients treated with alemtuzumab and over 62% of those receiving ocrelizumab demonstrated significant improvements in processing speed. However, neurologists must balance the potential advantages of escalating to high-efficacy disease-modifying therapies to prevent the progression of cognitive decline against the absence of evidence-based guidelines. In this context, sNfL, a biomarker reflecting ongoing axonal damage and uncontrolled disease activity, offers a promising tool for personalizing the management of cognitive impairment in MS [9,16].

In our study, we found that nearly 50% of the participants chose not to escalate to a higher-efficacy disease-modifying treatment in a simulated case of RRMS with progressive cognitive decline and pathologically elevated sNfL while receiving glatiramer acetate. The lack of full dedication to MS care and a limited perception of sNfL benefits were associated with DMEE in this hypothetical case.

Recent studies have demonstrated the existence of an association between sNfL and cognitive impairment (Table 3) [12,13,14,15,16,17,31,32]. A total of 127 patients with MS and 52 healthy controls were followed up for five years to assess physical and cognitive performances [14]. Patients with cognitive impairment exhibited higher sNfL levels compared to those without impairment. These elevated sNfL concentrations were linked to deficits in walking speed, manual dexterity, and processing speed. Additionally, baseline sNfL levels served as a predictor of processing speed. Another study examined the relationship between blood biomarkers of neurodegeneration and cognitive decline in a sample of 94 patients with MS at high risk of disease progression [15]. The findings revealed the existence of a significant correlation between sNfL and SDMT scores, with sNfL testing also predicting a decline in SDMT performance over three years. Van Dam et al. assessed the utility of a combination of imaging and fluid biomarkers to predict cognitive performance in a sample of 82 MS patients [17]. Fifty-six percent (n = 46) of the patients showed cognitive impairment, with a mean sNfL level of 10.54 pg/mL, compared to 8.45 pg/mL in the non-impaired group (*p* = 0.010). Serum and cerebrospinal fluid NfL levels were inversely correlated with processing speed. The most effective predictor of cognitive status was a multidimensional model that incorporated both sNfL levels and gray matter volume [13]. In line with these findings, another study showed that combining sNfL testing with magnetic resonance imaging markers, such as T2 hyperintense lesions and gray matter volumes, significantly enhances the accuracy of predicting cognitive impairment at the onset of the disease [12]. Higher sNfL levels were correlated with a poorer performance in the SDMT, indicating that early neuroaxonal loss is linked to cognitive dysfunction, particularly as regards processing speed. In addition, in a recent meta-analysis of 13 publications related to fluid biomarkers and cognitive impairment in MS, NfL was identified as the most effective marker [16]. The analysis found that higher sNfL levels were significantly associated with poorer processing speed scores, highlighting the relevance of sNfL as a marker of cognitive impairment in MS.

Our findings are consistent with previous research, highlighting how neurologists who are not exclusively dedicated to managing demyelinating diseases, compared to those specialized exclusively in MS, are more likely to make less optimal decisions regarding the initiation or intensification of disease-modifying treatments [33]. The current landscape of MS care has become increasingly complex due to the wide range of diagnostic and follow-up techniques, as well as the varied efficacy and safety profiles of the available treatments [22]. This evolving environment requires a comprehensive approach, integrating new treatments, outcome assessments, and biomarker utilization to enhance care delivery and patient outcomes [34]. As a result, it is challenging for general neurologists to stay abreast of the latest advances in managing cognitive impairment in MS. Patients with MS ideally should be managed by neurologists in specialized tertiary MS care centers, where expertise in clinical, radiological, and fluid biomarkers can be applied to the management of demyelinating diseases.

A recent study by Van Lierop et al. demonstrated the significant impact of sNfL testing on clinical decision-making among neurologists at an MS clinic in the Netherlands [35]. Clinical decisions were adjusted following the disclosure of sNfL results, particularly in the context of assessing new symptoms, performing differential diagnosis, and monitoring the efficacy of disease-modifying therapies. Higher sNfL levels were associated with an increased likelihood of decision changes. Furthermore, a moderate-to-high motivation to obtain sNfL results was observed in 54% of cases, underscoring clinicians’ interest in integrating sNfL data into patient care. The moderate recognition of the benefits of sNfL testing observed in our study can be attributed to several factors. These include the lack of specificity of the test, the absence of standardized measurement techniques, and the variation in reference ranges across different populations and neurological conditions [36,37]. Although efforts were made to minimize these issues by including a reference cut-off value for sNfL and clearly stating that no confounding factors affected the case scenario, they may still have influenced the responses.

We emphasize the importance of specialized MS care and the potential utility of sNfL as a biomarker to guide personalized treatment decisions to address the challenges identified in our study. Scientific societies play a critical role in this effort by disseminating knowledge, developing consensus guidelines, and organizing training programs to promote the use of emerging biomarkers like sNfL. Additionally, establishing standardized protocols for referring MS patients to specialized centers for advanced biomarker-based assessments may help to optimize treatment strategies and ensure the best possible care.

Our study has some limitations. First, the lack of practice guidelines for the prevention and treatment of cognitive impairment in MS care, aside from the various rehabilitation approaches available, may have influenced the decisions of the participants [3]. Second, we did not systematically assess prior knowledge about sNfL testing in MS and its potential usefulness in managing cognitive impairment. The lack of such knowledge could be a factor impacting the perceived usefulness of this assessment. Finally, focusing exclusively on participants from Spain and the relatively small sample size may limit the generalizability of our results. Future studies involving diverse cultural contexts, different health systems, and larger sample sizes may be necessary to validate our findings.

## 5. Conclusions

Cognitive impairment is a common and critical challenge in the management of RRMS. Our study found suboptimal decision-making among neurologists when faced with a simulated case of a clinically and radiologically stable patient experiencing cognitive decline with elevated sNfL levels, particularly among those not fully dedicated to MS care. The findings obtained emphasize the importance of specialized MS care and the potential utility of sNfL as a biomarker to guide personalized treatment decisions. Enhancing neurologists’ understanding and adoption of sNfL testing could improve clinical outcomes by facilitating timely and appropriate treatment adjustments for cognitive impairment in MS.

## Figures and Tables

**Table 2:** Multivariate logistic regression analysis.

	OR (95% CI)	*p*-Value
Age	0.86 (0.49–1.89)	0.74
Weekly MS patient load	1.0 (0.98–1.05)	0.49
Not full dedication to MS care	2.35 (1.01–5.50)	0.04
Low perception of sNfL benefits	1.02 (1.00–1.04)	0.01

CI, confidence interval; MS, multiple sclerosis; OR, odds ratio; sNfL, serum neurofilament light chain.

**Table 3:** Summary of previous research on cognition and sNfL in multiple sclerosis.

	*n*	Type of MS	Country
Jakimovski et al., 2020 [12]	127	85 RRMS, 42 progressive MS, 20 CIS	USA, Switzerland
Brummer et al., 2022 [14]	152	118 RRMS, 34 CIS	Germany
Barro et al., 2023 [15]	94	Not specified	USA
van Dam et al., 2023 [17]	46	28 RRMS, 3 PPMS, 12 SPMS, 2 CIS, 1 unknown	The Netherlands
Williams et al., 2022 [31]	110	110 SPMS	UK, Spain, Sweden, China
Mattioli et al., 2020 [32]	18	18 RRMS	Italy

CIS, clinically isolated syndrome; MS, multiple sclerosis; N, number of patients; PPMS, primary progressive multiple sclerosis; RRMS, relapsing–remitting multiple sclerosis; SPMS, secondary progressive multiple sclerosis.

## Data Availability

The datasets generated during the analysis of the study are available from the corresponding author on reasonable request.

## References

[1] Jakimovski D., Bittner S., Zivadinov R., Morrow S.A., Benedict R.H., Zipp F., Weinstock-Guttman B. (2024). Multiple sclerosis. Lancet.

[2] Benedict R.H.B., Amato M.P., DeLuca J., Geurts J.J.G. (2020). Cognitive impairment in multiple sclerosis: Clinical management, MRI, and therapeutic avenues. Lancet Neurol..

[3] De Meo E., Portaccio E., Bonacchi R., Giovannoli J., Niccolai C., Amato M.P. (2025). An update on the treatment and management of cognitive dysfunction in patients with multiple sclerosis. Expert. Rev. Neurother..

[4] Jellinger K.A. (2024). Cognitive impairment in multiple sclerosis: From phenomenology to neurobiological mechanisms. J. Neural Transm..

[5] Amato M.P., Prestipino E., Bellinvia A., Niccolai C., Razzolini L., Pastò L., Fratangelo R., Tudisco L., Fonderico M., Mattiolo P.L. (2019). Cognitive impairment in multiple sclerosis: An exploratory analysis of environmental and lifestyle risk factors. PLoS. ONE.

[6] Tur C., Portaccio E. (2024). Progression independent of relapse activity in multiple sclerosis: Time to account for cognitive decline. Mult. Scler..

[7] Honan C.A., Brown R.F., Batchelor J. (2015). Perceived cognitive difficulties and cognitive test performance as predictors of employment outcomes in people with multiple sclerosis. J. Int. Neuropsychol. Soc..

[8] Khalil M., Teunissen C.E., Lehmann S., Otto M., Piehl F., Ziemssen T., Bittner S., Sormani M.P., Gattringer T., Abu-Rumeileh S. (2024). Neurofilaments as biomarkers in neurological disorders—Towards clinical application. Nat. Rev. Neurol..

[9] Di Filippo M., Gaetani L., Centonze D., Hegen H., Kuhle J., Teunissen C.E., Tintoré M., Villar L.M., Willemse E.A., Zetterberg H. (2024). Fluid biomarkers in multiple sclerosis: From current to future applications. Lancet Reg. Health Eur..

[10] Freedman M.S., Gnanapavan S., Booth R.A., Calabresi P.A., Khalil M., Kuhle J., Lycke J., Olsson T. (2024). Guidance for use of neurofilament light chain as a cerebrospinal fluid and blood biomarker in multiple sclerosis management. EBioMedicine.

[11] Monreal E., Ruiz P.D., Román I.L.S., Rodríguez-Antigüedad A., Moya-Molina M.Á., Álvarez A., García-Arcelay E., Maurino J., Shepherd J., Cabrera Á.P. (2024). Value contribution of blood-based neurofilament light chain as a biomarker in multiple sclerosis using multi-criteria decision analysis. Front. Public Health.

[12] Jakimovski D., Zivadinov R., Ramanthan M., Hagemeier J., Weinstock-Guttman B., Tomic D., Kropshofer H., A Fuchs T., Barro C., Leppert D. (2020). Serum neurofilament light chain level associations with clinical and cognitive performance in multiple sclerosis: A longitudinal retrospective 5-year study. Mult. Scler..

[13] Ramani S., Berard J.A., Walker L.A.S. (2021). The relationship between neurofilament light chain and cognition in neurological disorders: A scoping review. J. Neurol. Sci..

[14] Brummer T., Muthuraman M., Steffen F., Uphaus T., Minch L., Person M., Zipp F., Groppa S., Bittner S., Fleischer V. (2022). Improved prediction of early cognitive impairment in multiple sclerosis combining blood and imaging biomarkers. Brain Commun..

[15] Barro C., Healy B.C., Saxena S., I Glanz B., Paul A., Polgar-Turcsanyi M., Guttmann C.R., Bakshi R., Weiner H.L., Chitnis T. (2023). Serum NfL but not GFAP predicts cognitive decline in active progressive multiple sclerosis patients. Mult. Scler..

[16] Rademacher T.D., Meuth S.G., Wiendl H., Johnen A., Landmeyer N.C. (2023). Molecular biomarkers and cognitive impairment in multiple sclerosis: State of the field, limitations, and future direction—A systematic review and meta-analysis. Neurosci. Biobehav. Rev..

[17] van Dam M., de Jong B.A., Willemse E.A.J., Nauta I.M., Huiskamp M., Klein M., Moraal B., de Geus-Driessen S., Geurts J.J.G., Uitdehaag B.M.J. (2023). A multimodal marker for cognitive functioning in multiple sclerosis: The role of NfL, GFAP and conventional MRI in predicting cognitive functioning in a prospective clinical cohort. J. Neurol..

[18] Preziosa P., Conti L., Rocca M.A., Filippi M. (2022). Effects on cognition of DMTs in multiple sclerosis: Moving beyond the prevention of inflammatory activity. J. Neurol..

[19] DeLuca J., Schippling S., Montalban X., Kappos L., Cree B.A., Comi G., Arnold D.L., Hartung H.P., Sheffield J.K., Liu H. (2021). Effect of Ozanimod on Symbol Digit Modalities Test Performance in Relapsing MS. Mult. Scler. Relat. Disord..

[20] Labiano-Fontcuberta A., Costa-Frossard L., Sainz de la Maza S., Rodríguez-Jorge F., Chico-García J.L., Monreal E. (2022). The effect of timing of high-efficacy therapy on processing speed performance in multiple sclerosis. Mult. Scler. Relat. Disord..

[21] Lechner-Scott J., Agland S., Allan M., Darby D., Diamond K., Merlo D., van der Walt A. (2023). Managing cognitive impairment and its impact in multiple sclerosis: An Australian multidisciplinary perspective. Mult. Scler. Relat. Disord..

[22] Saposnik G., Montalban X. (2018). Therapeutic Inertia in the New Landscape of Multiple Sclerosis Care. Front Neurol..

[23] Rodrigues R., Rocha R., Bonifácio G., Ferro D., Sabença F., Gonçalves A.I., Correia F., Pinheiro J., Loureiro J.L., Guerreiro R.P. (2021). Therapeutic inertia in relapsing-remitting multiple sclerosis. Mult. Scler. Relat. Disord..

[24] Melas C.D., Zampetakis L.A., Dimopoulou A., Moustakis V. (2012). Evaluating the properties of the Evidence-Based Practice Attitude Scale (EBPAS) in health care. Psychol. Assess..

[25] Hojat M., DeSantis J., Shannon S.C., Mortensen L.H., Speicher M.R., Bragan L., LaNoue M., Calabrese L.H. (2018). The Jefferson Scale of Empathy: A nationwide study of measurement properties, underlying components, latent variable structure, and national norms in medical students. Adv. Health Sci. Educ. Theory Pract..

[26] Courvoisier D.S., Cullati S., Haller C.S., Schmidt R.E., Haller G., Agoritsas T., Perneger T.V. (2013). Validation of a 10-item care-related regret intensity scale (RIS-10) for health care professionals. Med. Care.

[27] Dolan E.D., Mohr D., Lempa M., Joos S., Fihn S.D., Nelson K.M., Helfrich C.D. (2015). Using a single item to measure burnout in primary care staff: A psychometric evaluation. J. Gen. Intern. Med..

[28] Fuchs T.A., Schoonheim M.M., Zivadinov R., Dwyer M.G., Colato E., Weinstock Z., Weinstock-Guttman B., Strijbis E.M., Benedict R.H. (2024). Cognitive progression independent of relapse in multiple sclerosis. Mult. Scler..

[29] Bou Rjeily N., Mowry E.M., Ontaneda D., Carlson A.K. (2024). Highly Effective Therapy Versus Escalation Approaches in Early Multiple Sclerosis: What Is the Future of Multiple Sclerosis Treatment?. Neurol Clin..

[30] Labiano-Fontcuberta A., Monreal E., Benito-León J. (2022). Time to rethink the reported disease-modifying treatment effects on cognitive outcomes: Methods and interpretative caveats. Front. Neurol..

[31] Williams T., Tur C., Eshaghi A., Doshi A., Chan D., Binks S., Wellington H., Heslegrave A., Zetterberg H., Chataway J. (2022). Serum neurofilament light and MRI predictors of cognitive decline in patients with secondary progressive multiple sclerosis: Analysis from the MS-STAT randomised controlled trial. Mult. Scler..

[32] Mattioli F., Bellomi F., Stampatori C., Mariotto S., Ferrari S., Monaco S., Mancinelli C., Capra R. (2020). Longitudinal serum neurofilament light chain (sNfL) concentration relates to cognitive function in multiple sclerosis patients. J. Neurol..

[33] Saposnik G., Andhavarapu S., Fernández Ó., Kim H., Wiendl H., Foss M., Zuo F., Havrdová E.K., Celius E., Caceres F. (2022). Factors associated with treatment escalation among MS specialists and general neurologists: Results from an International cojoint study. Mult. Scler. Relat. Disord..

[34] Soelberg Sorensen P., Giovannoni G., Montalban X., Thalheim C., Zaratin P., Comi G. (2019). The Multiple Sclerosis Care Unit. Mult. Scler..

[35] van Lierop Z.Y., Wessels M.H., Lekranty W.M., Moraal B., Hof S.N., Hogenboom L., A de Jong B., Meijs N., A Mensing L., van Oosten B.W. (2024). Impact of serum neurofilament light on clinical decisions in a tertiary multiple sclerosis clinic. Mult. Scler..

[36] Moccia M., Terracciano D., Brescia Morra V., Castaldo G. (2024). Neurofilament in clinical practice: Is the multiple sclerosis community ready?. Mult. Scler..

[37] Lycke J., Fox R.J. (2024). Using serum neurofilament-light in clinical practice: Growing enthusiasm that may need bridling. Mult. Scler..

